# Unknown, Underserved, Underreported: A Case for Differentiation in Trauma Disorder Classification and Diagnosis

**DOI:** 10.7759/cureus.39157

**Published:** 2023-05-17

**Authors:** Hunter A Cutlip, Michael Ang-Rabanes, Raja Mogallapu

**Affiliations:** 1 Psychiatry, West Virginia University School of Medicine, Martinsburg, USA

**Keywords:** psychiatric diagnosis, dsm criteria, military psychology, complex-ptsd, ptsd diagnosis and treatment

## Abstract

This paper details the hospital course of a patient suffering from post-traumatic stress disorder (PTSD) who had been inadequately treated during previous hospitalizations and treatment programs. He also experienced symptoms not necessarily covered by the DSM-5 diagnosis of PTSD such as specific paranoia directed at his wife. This paper aims to expand upon the experiences of this patient from the standpoint of his disorder and his treatment history in order to demonstrate the potential benefits of the differentiation of complex PTSD (cPTSD) as a subset of patients within the greater scope of PTSD in order to more adequately address the needs of this subset of patients. Additionally, some common arguments against the recognition of cPTSD as a unique condition, such as diagnosing these patients as comorbid with bipolar disorder, are addressed.

## Introduction

Post-traumatic stress disorder (PTSD) is a psychiatric condition associated with intense, long-term mental and physical responses to past trauma. The National Institute of Mental Health estimates that the lifetime prevalence of PTSD is 6.8%, with a yearly prevalence of 3.6% among adults in the United States [1]. Up to 36.6% of PTSD patients are considered to be “severely impaired” on the Sheehan Disability Scale, underscoring an intense amount of functional impairment in the patient population. This is increasingly concerning as PTSD is associated with the physical effects of prolonged stress, increasing morbidity and mortality [2].

Patients with repeated or continuous trauma for an extended period of time may develop complex PTSD (cPTSD), which the ICD-11 defines as a patient who meets all qualifications of PTSD and demonstrates affect dysregulation, negative self-concept, and disturbances in relationships [3]. Conversely, cPTSD has not been included in the DSM due to a determination that there is not enough evidence for differentiation from traditional PTSD and overlap with borderline personality disorder [4]. The DSM declining to recognize cPTSD as a unique diagnosis means that psychiatric professionals are unlikely to recognize the condition and may, therefore, treat cPTSD patients as traditional PTSD cases. The discussion section of this paper assesses the literature on the effects of delineating these two conditions and is intended to demonstrate the net benefit to patient outcomes provided by addressing the factors unique to cPTSD.

This case report describes a patient with cPTSD who would have benefitted from closer follow-up and a more intense effort in initiating consistent outpatient therapy that would likely come with a stronger, specific diagnosis. By putting an emphasis on the compounded and difficult nature of this patient’s trauma, he may have been appropriately identified at an early stage, had significantly better outcomes, and potentially prevented suffering and extended hospitalizations in the future. In addition to delineating cPTSD from traditional PTSD, the presentation of this patient demonstrates the clinical necessity for the inclusion of cPTSD as a more severe subcategory of PTSD in further psychiatric treatment guidelines in order to assure standards of care are adequately met for this subset of patients.

## Case presentation

A 38-year-old male with the chief complaint of disorientation and auditory hallucinations presented to the ED, experiencing agitation and only oriented to self. His wife reported that she noticed he was becoming increasingly confused and more agitated than usual approximately one to two weeks prior to the presentation. The patient was not sleeping, was becoming very paranoid, and was preoccupied with religious punishment. The patient reported hearing voices accusing him of sin in association with his service and encouraging self-harm as retribution. The patient stated that he was prescribed eszopiclone 3mg and had been taking it for over a year: he reported that he overused it, ran out, and moved on to his wife’s identical prescription to no benefit. In order to ease his agitation regarding the ED's clinical assessment, the patient received 5mg haloperidol IM, 2mg lorazepam IM, and 50 mg diphenhydramine IM. The ED physician considered his initial presentation suspicious for schizophrenia or bipolar disorder. Urine drug screen at the time of admission is significant only for THC. His blood alcohol level was undetectable on admission.

The patient was admitted to an acute psychiatry unit for further evaluation and treatment. The initial assessment was significant for lack of a pressured speech. Thought processes vocalized on the first exam were primarily associated with voices as described above. Upon record review, this individual was found to have a past medical history of depression, PTSD, and anxiety. The patient was prescribed oxycodone/paracetamol 5-325mg for chronic back and joint pain associated with service and eszopiclone 3mg as a sleep aid from the Veterans Affairs (VA) but was not on medication for his anxiety or depression on admission. The patient reported no significant medication changes within the six months preceding admission. The patient had no relevant family history. Psychiatric history was positive for several hospitalizations for similar psychiatric events but had not been followed in an outpatient setting as of present admission.

The patient’s social history included the completion of a tour of duty as a member of the Army, during which he endured repeated instances of artillery fire over a period of several months. During treatment, the patient expressed feelings of guilt regarding his actions overseas. He was unemployed due to an inability to maintain consistent employment in his field due to his expressed symptomatology. Per his family, the patient has a history of alcohol use disorder, and his drinking intensified a baseline level of concern and anxiety that the family feels from being around him. They noted that he had two beers at a recent holiday party and had been sober since; the patient denied any additional substance use or medication abuse.

Upon initial presentation and evaluation by psychiatry, bipolar spectrum and schizophrenic disorders were considered in addition to an exacerbation of PTSD. A review of the psychiatric history and discussion with the patient did not elucidate any manic or hypomanic episodes. The patient did not endorse any manic symptoms during this episode. Bipolar disorders were ruled out on this basis. The initial presentation was suspicious of schizophrenia due to delusions. The patient's presenting symptoms supported a schizophrenia or schizoaffective disorder; however, due to the significance of his military service as the focus of many of his symptoms, PTSD was considered a primary contributing factor for presentation.

The patient was prescribed 2mg risperidone twice a day for acute psychosis and 1mg prazosin for night terrors. His baseline therapy was augmented with haloperidol 5mg as needed. Non-pharmacologic treatment included encouragement to attend daily group therapy. A few days after admission, the patient developed jaw dystonia concerning for onset of extrapyramidal side effects. He was immediately transitioned from risperidone to 5mg olanzapine at night in order to prevent the progression of dystonia.

During his stay, the risperidone and olanzapine appeared to improve his auditory hallucinations, and he reported they were becoming less frequent. His affect progressed from flat to restricted, as he was continually suspicious of medical personnel on the unit. Furthermore, he was consistently positive for delusions of being in a large-scale experiment and thought to broadcast (the patient-reported belief that his thoughts were audible or otherwise known to others). However, the patient remained medication compliant and agreed to begin individual, trauma-focused cognitive therapy. During his hospitalization, he participated in daily sessions. The patient began to gain trust in the medical team despite a short period of regression during a transition of care between attending physicians.

Over the course of an extended stay on the unit, the patient began to understand his disposition and was able to talk about his past history of PTSD and depression for the first time. He completed the International Trauma Questionnaire and met their criteria for cPTSD. This questionnaire result in addition to the patient’s expansion on the severity and depth of his trauma was sufficient to further diagnose the patient with cPSTD. The patient’s eszopiclone and oxycodone were tapered and discontinued due to concerns that they may have precipitated his altered mental status in part. Olanzapine was titrated to 20mg prior to discharge. At that time, it was determined that the patient had achieved adequate control of his symptoms including mood, affect, hyper-vigilance, and avoidance and was considered safe for discharge. He was able to vocalize relief from cognitive distortions related to the trauma he had sustained. The patient was scheduled for outpatient follow-up. The patient was lost to follow-up as he returned to the VA for treatment of his condition.

## Discussion

The patient presented in this report demonstrated severe symptoms of PTSD and interpersonal issues on admission. Through the course of his admission, his additional symptoms of distrust, insomnia, and hypervigilance justify a further diagnosis of cPTSD as outlined in this discussion. The patient’s known history of trauma is relatively brief compared to other patients with cPTSD (a tour of duty in the American military averages 8 months) [5]. His continuous exposure to severe stress during this time, his stated guilt relating to his actions during his service, and the potential of inappropriate follow-up to be a contributing factor to his lack of trust aided in meeting the criteria for a final diagnosis of PTSD. Shared symptoms more common in cPTSD, such as substance use and aggression, were also present in this case. A diagnosis of borderline personality disorder (BPD) was considered inappropriate in this case when considering the patient’s overall clinical picture. While the patient could be considered to meet BPD diagnostic criteria per the broader symptom characteristics described in the DSM-5, this patient failed to meet the diagnostic criteria for BPD. Additionally, symptoms demonstrated by the patient that would indicate a BPD diagnosis are better explained by PTSD with inadequate treatment. Therefore, cPTSD is the most reasonable diagnosis for this patient.

cPTSD is not a currently recognized diagnosis in the DSM-5. However, considering this patient’s symptoms at presentation, it is appropriate in this case. First described in 1992 by Dr. Judith Herman, cPTSD presents with more severe forms of the symptoms of PTSD, as well as some additional conditions associated with the repetitive nature of the trauma. The criteria established for the diagnosis included the following five areas: behavioral (substance abuse and self-destructive behaviors), emotional (lability, depression, and anger management issues), cognitive (dissociation and identity conflicts), interpersonal (difficulty with trust and difficulties maintaining interpersonal relationships), and somatization. While many of these symptoms are present in PTSD, patients with cPTSD are more likely to experience them and often have much more severe presentations. Thus, Dr. Herman characterized cPTSD as a condition that “may coexist with simple PTSD but extends beyond it” [6].

The lack of formally outlined diagnostic criteria or other forms of definition for cPTSD in the DSM-5 is likely to have an adverse effect on the treatment of this subset of patients as the unique features of their disease may fail to be recognized and treated. For instance, cPTSD patients appear to have a higher prevalence of somatization symptoms relative to other PTSD patients, which in turn leads to increased utilization of medical services [2]. While cPTSD has limited data regarding physical manifestations, the mechanism and presentation are the same as traditional PTSD. The effects of chronic stress increase the relative risk of hypertension, myocardial infarction, and stroke [7]. In addition, psychosomatic manifestations of PTSD can include chronic pain, fibromyalgia, and irritable bowel syndrome. Some studies suggest that the source of these pain syndromes in affected patients can be traced to diagnosis-associated hypothalamic-pituitary axis changes [2].

Similarities have been noted between borderline personality disorder and the defining symptoms of cPTSD, and it has been suggested that cPTSD is simply a PTSD patient with comorbid BPD [4]. Both have etiology heavily associated with childhood trauma and are associated with impulsive, self-destructive tendencies, feelings of anger, and paranoia. It should be noted that many of these similarities are common root causes or symptoms among several psychiatric diagnoses. Similar to these established conditions, it is the unique combination and clinical picture of the disorder that should be used to discriminate between cPTSD and BPD. Furthermore, there are several key differences in the presentation of these patients. Both BPD and cPTSD have symptoms of hypervigilance in their interpersonal relationships. However, an analysis of the underlying cause of this symptom reveals a core difference in the emotional source of the symptoms. BPD patients are likely hypervigilant due to an underlying fear of external invalidation, such as through shame or abandonment. [8] In contrast, cPTSD patients are likely responding to an internal fear of bodily harm as a subconscious defense mechanism resulting from trauma. Finally, the biochemical pathway dysregulation associated with BPD appears to originate in the anterior cingulate cortex and prefrontal cortex. These changes have not been demonstrated in studies of cPTSD patients to date [4].

Studies regarding the treatment of cPTSD provide evidence that treatment is similar to the standard of care treatment for PTSD with some distinct variance in requirements and overall effectiveness. A 2021 study comparing PTSD and cPTSD patients demonstrated that patients who have been diagnosed with cPTSD did not have statistically significant differences in response to cognitive therapy-based treatment despite a higher baseline severity of the condition as measured by CAPS-5 score [9]. The efficacy of traditional PTSD treatment in this patient population demonstrates that an eventual standard of care is likely to incorporate CBT in some way. However, it should be noted that while the trajectory of patient response to treatment was similar in the PTSD and cPTSD populations, the latter group had a higher CAPS-5 score at every measured time interval. Further research is required in order to determine if there is a regimen that increases treatment response for cPTSD patients and whether this differs in intensity, content, or frequency. For example, all of the patients in the aforementioned study were seen once per week. Frequent visits may be found to be beneficial for alleviating trust issues in this subset of patients which more commonly present with such symptoms. As demonstrated in our case report, the required increase in visit frequency is not directly evident in the more severe nature of the associated trauma if the condition is simply diagnosed as a more severe presentation of traditional PTSD. This lack of acknowledgment of the unique needs of a cPTSD patient necessarily leads to inadequate and substandard psychiatric treatment. In order to ensure appropriate management of harmful outcomes in cPTSD, a separate diagnosis is crucial.

The treatment of cPTSD as a variant of PTSD with comorbid BPD has the potential to demonstrate falsely inflated treatment results due to symptom control without adequate management of the underlying root cause of the disorder. While the cognitive behavioral therapy (CBT) regimen for PTSD has shown efficacy in cPTSD patients as would be expected, the issue arises when considering dialectical behavioral therapy (DBT) in these patients per APA guidelines for the treatment of BPD [10]. If these patients are on a typical treatment frequency for a patient with PTSD, they may not be optimally addressing the source of their trauma. This would likely result in a demonstrable treatment failure, which could lead to increased visits or a change in approach. However, DBT is designed to help patients manage their emotions and take responsibility for the thought patterns that lead to the emotions and behaviors that they exhibit. Research into the standard of care has not been sufficient to date, largely due in part to case ascertainment secondary to a lack of DSM definitional clarity. The effects of DBT must then be examined in the interim based on a theoretical risk-benefit analysis. More specifically, the effects DBT may have on the self-blame tendencies of a PTSD patient are particularly relevant in this assessment. Patients who demonstrate tendencies to blame themselves for their trauma and its resultant effect on their lives are at higher risk of having severe symptoms [11]. A study of predictive factors for improvement in PTSD symptoms demonstrated that a reduction in the tendency for a patient to blame themselves was associated with an improvement in symptoms [12]. cPTSD patients are likely to have a higher risk for developing self-blaming coping mechanisms by means of having inherently more severe PTSD symptomatology at presentation. As noted above, many patients with cPTSD are instead considered PTSD patients with comorbid BPD. Treatment guidelines, therefore, suggest that these patients would benefit from spending significant amounts of their treatment time in DBT. However, DBT would not address these issues of self-blame and in this patient base only serves to modulate the expression of symptoms via the regulation of distress tolerance responses. This produces the mechanism by which treatment response is inappropriately evaluated. Without appropriate study into the cPTSD patient base, such fundamental issues in the assessment of treatment create the risk of perpetuating an inappropriate and under-appreciated failure of treatment. Patient safety and well-being are threatened by a failure to develop an academic and clinical environment for the development of evidence-based medicine for such a severely at-risk population. Until further evidence-based practice can be elucidated from the community at large, the psychiatric community should be particularly mindful of the treatment techniques used in patients suspected to have some of the more severe traumatic responses associated with a potential diagnosis of cPTSD.

Critics of the distinction of cPTSD have stated that it is unprecedented to grant a distinct diagnosis to a condition that is essentially a more severe form of a previously established diagnosis [13]. However, it can be argued within the context of trauma response that precedent has been established within the DSM on the topic of stress disorders demonstrates that a disorder need not necessarily be concretely unique in order to be differentiated from similar conditions. Patients could not be diagnosed with PTSD until symptoms had persisted for at least a month. In order to provide appropriate coverage and treatment for patients with the intent to divert them from a more serious condition, the DSM-IV introduced Acute Stress Disorder (ASD) as a diagnosis in 1994 [14]. This is a clear example of a diagnosis added for the express purpose of directing clinical management. Changes made to PTSD and ASD in the DSM-5 further solidified their relationship and created a de facto spectrum of trauma responses. ASD no longer has a specific focus on dissociative symptoms and is now solely diagnosed based on timing, the presence of Criterion A PTSD symptoms, and a number of symptoms specific to the condition. It is within this framework established by the history of ASD that cPTSD should be considered for future inclusion in the DSM [15].

Including cPTSD in the established stress disorder spectrum recognizes arguments regarding the overlap of these conditions while equally acknowledging the unique symptomatology of the condition and encouraging further study of outcomes in these patients [16]. Providing a consistent definition for psychiatric study for this subset of patients would improve the validity of research and consequently the strength of resulting recommendations. The data on cPTSD treatment in relation to PTSD, while limited, does demonstrate some clinical differences from a traditional diagnosis. Perceived social support has been identified as a significant factor in many ICD-11 diagnosed cPTSD cases as assessed by the International Trauma Questionnaire (ITQ) and may be a potential avenue for therapeutic intervention [17]. The ITQ provides a screening tool for PTSD based on the presence of one of the following two symptoms: reliving traumatic experiences or avoidance of triggers associated with these experiences. These must be accompanied by a "sense of current threat" and create functional impairment. A further diagnosis of cPTSD may be met if the previous criteria are accompanied by "disturbances of self-organization" symptoms such as affective dysregulation, disruption of personal relationships, and negative self-concept [18]. The questionnaire is designed to address these questions through their impact on the patient's life and ability to function.

Significant studies contrasting treatment duration between PTSD and cPTSD do not appear to have been performed to date due to the relatively new definition of the latter condition but are likely to demonstrate an inherent difference due to the nature of the trauma and the constellation of additional symptoms potentially requiring a mixed approach to therapy. Treatment guidelines regarding the benefits of medications alone or in addition to therapy are unclear at this time.

## Conclusions

cPTSD is a unique clinical presentation that shares features with several other psychiatric conditions. The severity and presence of symptoms which can be directly linked to the prolonged or continuous nature of the inciting trauma should be used to differentiate from the current DSM definition of PTSD. Simply considering cPTSD as PTSD with concomitant borderline personality disorder fails to account for the key differences in presentation between the two conditions.

Ultimately, the case for cPTSD as a distinct diagnosis is contingent on the necessity of the modification of clinical decision-making associated with this subset of patients. Failing to consider the severity of the condition or the challenges to treatment inherent in traumatic conditions, compounded with difficulties with trust often experienced in this condition predisposes such cases to inadequate treatment and worse patient outcomes. As illustrated in the case presented here, a blanket definition of PTSD is insufficient for identifying those patients who would benefit from additional resource allocation and increased surveillance associated with a more specific diagnosis.
